# Relaxin as a Therapeutic Target for the Cardiovascular Complications of Diabetes

**DOI:** 10.3389/fphar.2018.00501

**Published:** 2018-05-15

**Authors:** Hooi Hooi Ng, Chen Huei Leo, Laura J. Parry, Rebecca H. Ritchie

**Affiliations:** ^1^School of BioSciences, The University of Melbourne, Melbourne, VIC, Australia; ^2^Heart Failure Pharmacology, Baker Heart and Diabetes Institute, Melbourne, VIC, Australia; ^3^Department of Human and Molecular Genetics, Herbert Wertheim College of Medicine, Florida International University, Miami, FL, United States; ^4^Science and Math Cluster, Singapore University of Technology and Design, Singapore, Singapore; ^5^Department of Pharmacology & Therapeutics, The University of Melbourne, Melbourne, VIC, Australia

**Keywords:** relaxin, diabetes, vasculopathy, endothelial dysfunction, remodeling, cardiomyopathy

## Abstract

Cardiovascular complications are the major cause of mortality in patients with diabetes. This is closely associated with both macrovascular and microvascular complications of diabetes, which lead to organ injuries in diabetic patients. Previous studies have consistently demonstrated the beneficial effects of relaxin treatment for protection of the vasculature, with evidence of antioxidant and anti-remodeling actions. Relaxin enhances nitric oxide, prostacyclin and endothelium-derived hyperpolarization (EDH)-type-mediated relaxation in various vascular beds. These effects of relaxin on the systemic vasculature, coupled with its cardiac actions, reduce pulmonary capillary wedge pressure and pulmonary artery pressure. This results in an overall decrease in systemic and pulmonary vascular resistance in heart failure patients. The anti-fibrotic actions of relaxin are well established, a desirable property in the context of diabetes. Further, relaxin ameliorates diabetic wound healing, with accelerated angiogenesis and vasculogenesis. Relaxin-mediated stimulation of vascular endothelial growth factor (VEGF) and stromal cell-derived factor 1-α, as well as regulation of metalloproteinase expression, ameliorates cardiovascular fibrosis in diabetic mice. In the heart, relaxin is a cardioprotective molecule in several experimental animal models, exerting anti-fibrotic, anti-hypertrophy and anti-apoptotic effects in diabetic pathologies. Collectively, these studies provide a foundation to propose the therapeutic potential for relaxin as an adjunctive agent in the prevention or treatment of diabetes-induced cardiovascular complications. This review provides a comprehensive overview of the beneficial effects of relaxin, and identifies its therapeutic possibilities for alleviating diabetes-related cardiovascular injury.

## Introduction

Diabetes mellitus is a major risk factor for the development of cardiovascular diseases. This disease affects an estimated 6.4% of adults worldwide, but this number is likely to increase to 7.7% by 2030 (Shaw et al., 2010). Diabetes leads to microvascular complications such as kidney failure, lower limb amputation, and blindness (Forbes and Fotheringham, 2017). Macrovascular complications of diabetes include peripheral vascular disease, myocardial infarction, stroke, and congestive heart failure. In the context of diabetic cardiomyopathy, left ventricular diastolic dysfunction at an early stage which subsequently progress to systolic dysfunction is closely associated with cardiac hypertrophy and fibrosis (Boudina and Abel, 2007). We and others have reviewed the key mechanisms and strategies to target diabetic cardiomyopathy (Boudina and Abel, 2010; Huynh et al., 2014; Ritchie et al., 2017; Tate et al., 2017b). It is however worth noting that there are still no effective therapies to limit the progression of diabetes-induced cardiovascular complications. Here, we propose a small peptide hormone, relaxin, which shows a promising role in protecting the cardiovascular system (Leo et al., 2016a; Sarwar et al., 2017). The possibilities of relaxin as a therapy for diabetes-related complications are also discussed.

## Cardiovascular Complications of Diabetes

Diabetic cardiomyopathy is characterized by damage to the myocardium, in particular diastolic dysfunction (Pappachan et al., 2013), where the heart is unable to relax and undergo filling during the diastolic part of the cardiac cycle. Pathological processes underlying this include cardiac hypertrophy, neuronal abnormalities and myocardium stiffening as a result of collagen cross-linking and extracellular matrix deposition (Iimoto et al., 1988). Activation of the sympathetic nervous system as a result of insulin resistance leads to impaired kidney and vascular function, an effect that is associated with the onset of hypertension (Reaven et al., 1996). For example, an FDA-approved glucose-lowering drug for the treatment of type 2 diabetes, the sodium-glucose co-transporter 2 (SGLT2) antagonist empagliflozin, exhibits promising actions in reducing cardiovascular burden by limiting the sympathetic outflow to the kidneys (Sano, 2018). Diabetes-induced collagen cross-linking that accumulates within the myocardium can ultimately lead to structural remodeling of the heart tissue, including cardiac fibrosis. Mechanisms that contribute to the phenotype of the diabetic myocardium include (but are not limited to) impairments in function of type 2 ryanodine receptors (RyR2) (Santulli et al., 2015), increased oxidative stress, activation of the renin-angiotensin-aldosterone system (RAAS), endothelin-1 upregulation, with mitochondrial dysfunction, inflammation and endoplasmic reticulum stress (Huynh et al., 2014; Prakoso et al., 2017; Tate et al., 2017a,b). Reactive oxygen species (ROS) directly damage proteins, DNA and lipids, produce reactive lipid peroxidases, and can generate reactive nitrogen species, all which contribute to diabetic cardiomyopathy (Baynes and Thorpe, 1999). In diabetes, the over-activation of the RAAS increases angiotensin II (Ang II) production in cardiac fibroblasts and cardiomyocytes, to impair cardiac structure and function (Lavrentyev et al., 2007; Singh et al., 2008). Similarly, these pathological pathways also play a major role in diabetes-associated vasculopathy. The most well-known functional characteristic of diabetes-induced vascular complications is endothelial dysfunction, where there is an imbalance in vascular homeostasis, causing a shift toward vasoconstrictor state and reduced vasodilation. Hyperglycemia leads to the imbalance of vasoprotective molecules such as nitric oxide (NO), prostacyclin (PGI_2_), and endothelium-derived hyperpolarization (EDH) pathways, and causes overproduction of ROS or vasoconstrictor, thus leading to endothelial dysfunction (Johnstone et al., 1993; Guzik et al., 2002; Angulo et al., 2003; Vanhoutte et al., 2017). Both the cardiac (Bugger and Abel, 2014; Huynh et al., 2014; Tate et al., 2017b) and vascular complications (Forbes and Fotheringham, 2017; Shi and Vanhoutte, 2017) of diabetes have been extensively reviewed in detail elsewhere. Insulin resistance can be initiated by altered glucose metabolism and disturbance in fatty acid metabolism (McGarry, 1992), whereby there is an imbalance between fatty acid uptake and beta-oxidation in the skeletal muscle or heart, leading to impaired insulin signaling. Inhibition of both glucose metabolism and fatty acid uptake (Randle et al., 1963), and muscle glucose uptake via PKCζ activation (Samuel and Shulman, 2016) all contribute to the pathophysiology of diabetes.

## Is Glucose-Lowering Sufficient?

Insulin therapy is the first line treatment for type 1 diabetic patients. One school of thought favors a role for insulin therapy in heart failure outcomes, particularly in the elderly (Sardu et al., 2014). However, this is countered by evidence from several studies in which diabetic patients on insulin therapy exhibit increased risk of developing heart failure (Nichols et al., 2005; Smooke et al., 2005; Mangiavacchi et al., 2008). Over the years, many approaches to safely treat type 2 diabetes have been developed. First-line glucose-lowering therapy for type 2 diabetes remains to be metformin (Holman, 2007). Recently, several new classes of drugs have been developed for treating type 2 diabetes, which include dipeptidyl peptidase-4 (DPP4) inhibitors, glucagon-like peptide-1 (GLP-1) receptor agonists and SGLT2 antagonists. These therapies can be added to first-line therapy if adequate glycemic control is not yet achieved, aiming to reduce and control blood glucose levels for as long as possible after diagnosis, and thereby potentially preventing the development and progression of cardiovascular complications. However, the majority of these conventional glucose-lowering drugs fail to prevent the progression of cardiovascular complications, despite their favorable effects on glycemic control. There has recently emerged the suggestion that GLP-1 receptor agonists may reduce cardiovascular complications, although this may be secondary to the reduction in glycated hemoglobin levels (Bethel et al., 2017). Excitingly however, the SGLT2 inhibitors empagliflozin and canagliflozin reduce heart failure hospitalization and may be the first glucose-lowering therapy to relieve some of the cardiovascular burden of diabetes as demonstrated in the EMPA-REG OUTCOME and CANVAS trials (Lytvyn et al., 2017). Hence, alternative therapies are needed to address the ever-increasing incidence of diabetes to prevent and/or treat diabetes-induced cardiovascular complications.

## Protective Role for Relaxin in the Vasculature

An emerging molecule implicated in the protection of the cardiovascular system is the hormone relaxin. In rodents, the relaxin/insulin-like family peptide receptor 1 (RXFP1) is expressed in the aorta (Ng et al., 2015), small renal and mesenteric arteries (Novak et al., 2006). RXFP1 is predominantly localized in the endothelial cells of rat mesenteric artery and vein, vena cava and the aorta, but the distribution of this receptor is more abundant in the vascular smooth muscle cells of rat small pulmonary artery, femoral artery and vein (Jelinic et al., 2014). The differential distribution of RXFP1 in several vascular beds could serve as an important target for relaxin activity independent of any potential source of local relaxin production. The other members of the relaxin family peptide receptors comprise RXFP2, RXFP3, and RXFP4. Relaxin can weakly bind to and activate RXFP2 at higher concentrations, however, in human and rodents, relaxin has higher binding affinity to RXFP1. RXFP3, and RXFP4 on the other hand are structurally and functionally different to RXFP1 and RXFP2, with their corresponding ligands relaxin-3 and INSL5 (Bathgate et al., 2006). Traditionally, relaxin is known as a reproductive hormone that plays an important role in maternal adaptations to pregnancy, by elongating the pubic ligament at the end of pregnancy in rodents, as well as causing cervical ripening and nipple development (Sherwood, 2004). However, many actions of relaxin have also been extensively reported in several non-reproductive organs (Leo et al., 2016a; Samuel et al., 2016; Sarwar et al., 2017). Much of this has relied on the potent anti-fibrotic actions of relaxin (Samuel et al., 2016). Exogenous relaxin administration also has significant therapeutic benefits in animal models of hypertension, atherosclerosis and tobacco-related disease, often beyond its anti-fibrotic mechanisms, as detailed below.

In the vasculature, relaxin reduces myogenic reactivity and sensitivity to the α1-adrenergic vasoconstrictor, phenylephrine in Wistar Kyoto rats (WKY) and spontaneously hypertensive rats (SHR), accompanied by improved flow-mediated vasodilation (van Drongelen et al., 2013). This action is NO-dependent in normotensive, but not hypertensive rats (van Drongelen et al., 2013). In Ang II-induced hypertensive rats, relaxin reduces mean arterial pressure, albumin excretion and impairments in NO availability, effects attributed to reduced oxidative stress (Sasser et al., 2011). Furthermore, relaxin attenuates aortic remodeling to reduce wall thickness and collagen content (Xu et al., 2010). Inward remodeling of parenchymal arterioles is also ameliorated in hypertensive rats (Chan et al., 2013), thus increasing arterial compliance. These studies in hypertensive models clearly show the vasculoprotective effects of relaxin against hypertension-induced dysfunction and its potential role in reversing hypertension-associated fibrosis. More recently, relaxin treatment is reported to reverse cigarette smoke-induced endothelial dysfunction, an effect attributed to enhanced endothelial NO synthase (eNOS) expression (Pini et al., 2016). Relaxin infusion over 4 weeks also reverses the atherosclerosis-associated endothelial dysfunction in *ApoE^-/-^* mice, an effect attributed to reduction in angiotensin AT1 receptors and oxidative stress (Tiyerili et al., 2016). In addition, relaxin-mediated vasculoprotective effects are also evident in vascular preparations *ex vivo*, primarily through its action on endothelin B receptors (ET_B_), NO and PGI_2_ (Dschietzig et al., 2003; McGuane et al., 2011a; Dschietzig et al., 2012; Ng et al., 2016). In isolated small human gluteal (Fisher et al., 2002) and subcutaneous (McGuane et al., 2011b) arteries, relaxin induces rapid dilator actions. However, the rapid vasodilator effects of relaxin are not observed in all blood vessels such as the rat mesenteric arteries, despite the presence of RXFP1 (Leo et al., 2014b, 2016a, 2017). All of these studies provide an important insight into the protective mechanisms of relaxin against vascular dysfunction, where altered vasodilator and vasoconstrictor pathways are implicated, as well as modifying the vessel wall structure.

## Cardiac Role for Relaxin

In addition to the above vasoprotective roles of relaxin, a plethora of literature has identified relaxin as a cardioprotective molecule, particularly through its anti-fibrotic and anti-hypertrophic actions. For example, relaxin ameliorates post-infarction fibrotic healing by reducing the density of mature scar tissue in the infarcted area (Samuel et al., 2011), and regulates cardiac fibroblast proliferation, differentiation and collagen deposition, thereby reversing cardiac fibrosis *in vivo* (Samuel et al., 2004). In SHR, relaxin administration for 2 weeks is sufficient to lower collagen content in the left ventricle, ameliorate left ventricular hypertrophy and dysfunction, as well as reverse cardiac fibrosis (Lekgabe et al., 2005). Furthermore, relaxin also prevents atrial fibrillation secondary to the amelioration of cardiac fibrosis and hypertrophy in SHR. This action is associated with changes in Na^+^ current density (Parikh et al., 2013; Henry et al., 2016). One of the well-reported mechanisms of relaxin as an anti-fibrotic molecule is its ability to up-regulate the Notch signaling pathway, whereby the transforming growth factor (TGF)-β/Smad 3 signaling is inhibited to prevent cardiac fibroblast-myofibroblast transition (Sassoli et al., 2013). In addition to the potent anti-fibrotic actions of relaxin in various disease animal models, relaxin has the ability to reduce oxidative stress, at least in part via protein kinase B (Akt) and extracellular signal-regulated kinase (ERK) signaling pathways, suppressing cardiomyocytes apoptosis and hypertrophy (Moore et al., 2007). Collectively, the actions of relaxin in pressure-overloaded animals may represent an attractive target of unloading the heart, via vasodilation and reducing systemic vascular resistance.

## Role for Relaxin in the Context of Diabetes-Induced Vascular Complications

Further to the widely reported actions of relaxin in hypertensive animals, several lines of evidence suggest that relaxin may possess similar effects in diabetic animals. Relaxin ameliorates diabetic wound healing by accelerating angiogenesis and vasculogenesis through the stimulation of vascular endothelial growth factor (VEGF) and stromal cell-derived factor (SDF)1-α, as well as regulating metalloproteinase (MMP) expression to impede fibrosis in *db/db* mice (Bitto et al., 2013; Squadrito et al., 2013). These wound healing properties of relaxin are primarily attributed to induction of VEGF expression and subsequent angiogenesis that selectively target the wound site (Unemori et al., 2000). Relaxin also upregulates NO production in the vasculature, which may play an additional role in wound healing. Notably, relaxin treatment attenuates fibrosis in the heart of streptozotocin (STZ) Ren-2 diabetic rats by decreasing interstitial and total left ventricle collagen deposition, thereby reducing myocardial stiffness and improving overall left ventricular diastolic function (Samuel et al., 2008). Indeed, relaxin treatment inhibits both proliferation of cardiac fibroblasts and formation of type I and III fibrillar collagen induced by high glucose *in vitro* (Wang et al., 2009). Intriguingly, relaxin also been reported to attenuate skeletal muscle insulin resistance, accompanied by enhanced aortic endothelium-dependent relaxation in high fat-fed pre-diabetic mice (Bonner et al., 2013). The underlying mechanisms of relaxin actions in this context however are not further interrogated.

Our own studies have recently demonstrated that relaxin protects the mouse aorta and mesenteric arteries by restoring endothelial function under acute (high glucose) (Ng et al., 2016) and chronic hyperglycemia (Ng et al., 2017). Relaxin prevents high glucose-induced endothelial dysfunction in mouse aorta by suppressing oxidative stress and stimulating vasodilator PGI_2_ production, as well as changing the relative gene expression of the PGI_2_ receptor (IP) to thromboxane (TP) (Ng et al., 2016). Under high glucose conditions, PGI_2_, which normally acts as a vasodilator, reverts to a vasoconstrictor (Zhu et al., 2014), likely acting through the TP receptor. This *ex vivo* study on relaxin co-treatment under hyperglycemic conditions provided ‘proof-of-concept’ evidence that motivated us to further investigate the *in vivo* effects of relaxin utilizing chronic type 1 diabetic mice. It is well-established that chronic hyperglycemia induces endothelial dysfunction in an experimental model of diabetes (Pieper et al., 1995; Leo et al., 2011a), with downregulation of endothelium-derived NO. Consistent with the *ex vivo* data, relaxin treatment for 2 weeks following 10 weeks of untreated diabetes is sufficient to restore endothelial function in diabetic mouse aorta (Ng et al., 2017). This is underpinned by enhanced NO-mediated relaxation. Interestingly, relaxin treatment in this context failed to increase basal NO availability in both *ex vivo* and *in vivo* settings of hyperglycemia. This suggests that exogenous relaxin treatment selectively augments agonist stimulated NO-mediated relaxation pathways to preserve endothelial vasodilator function, without impacting on basal NO synthase activity in endothelial cells. In contrast to the findings in diabetic aortae, relaxin increases basal NOS activity and eNOS phosphorylation in healthy rat aortae following two days of continuous intravenous infusion (Leo et al., 2016b), leading to enhanced NO-mediated relaxation. The disparity of findings may be attributed to the difference between non-diabetic and diabetic settings, or deficits in the NOS co-factor, tetrahydrobiopterin (BH_4_), in diabetic aortae, as a result of oxidation by peroxynitrite produced from the interaction between superoxide and NO in the endothelium (Cai et al., 2005).

Diabetes also attenuates endothelial function in smaller resistance arteries (mesenteric arteries), by increasing the contribution of vasoconstrictor prostanoids to endothelial dysfunction and reducing EDH-type relaxation (Ng et al., 2017). It is worth noting that the role of EDH increases as the artery diameter decreases, thus the relaxation seen in mesenteric artery will be largely dependent on EDH, in contrast to larger blood vessel such as the aorta. Relaxin treatment for 2 weeks in diabetic mice reverses endothelial dysfunction (Ng et al., 2017). This is accompanied by a profound increase in the relative contribution of NO to endothelium-dependent relaxation. Although diabetes selectively impairs EDH-type relaxation in the mesenteric artery, relaxin treatment failed to restore EDH-type relaxation at least in the STZ C57BL/6 mouse model of diabetes. The mechanisms underpinning the impairment of EDH-type relaxation in diabetic mesenteric arteries are multi-factorial (Ding et al., 2005; Ding and Triggle, 2010; Leo et al., 2011b). One possible explanation is that relaxin is not able to restore the reduction in low-resistance electrical coupling between endothelial and vascular smooth muscle cells in the mouse small mesenteric arteries in the context of diabetes (Ding et al., 2005). Indeed, the mechanism of relaxin action is quite distinct to that of NO in reversing diabetes-induced endothelial dysfunction, possibly by reducing superoxide production and increasing eNOS dimerization. Relaxin treatment for two to five days in healthy rats significantly enhances NO-mediated relaxation in the mesenteric artery (van Drongelen et al., 2011, 2012, 2013; Jelinic et al., 2014; Leo et al., 2016b). Despite compelling evidence to suggest that relaxin treatment augments EDH-type relaxation in mesenteric arteries (Leo et al., 2014b) and cerebral parenchymal arterioles (Chan and Cipolla, 2011), with activation of intermediate conductance Ca^2+^-activated K^+^ channels, it is worth mentioning that these experiments were done in healthy male and non-pregnant female rats. Therefore, the discrepancies in animal species (mice vs. rats) and physiological conditions (diseased vs. healthy) may account for the differences in the mesenteric artery response to exogenous relaxin.

A key striking effect of relaxin treatment in diabetic mice is the normalization of vasoconstrictor prostanoids in the mesenteric artery. Consistent with this, our group previously shown that *Rln^-/-^* mice have altered prostanoid pathways in both the mesenteric artery (Leo et al., 2014a) and aorta (Ng et al., 2015). We suggest that relaxin likely restores diabetes-induced endothelial dysfunction by affecting the NO and prostanoid pathways, but not the EDH pathway, in both the aorta and mesenteric artery. In addition to these actions of relaxin in regulating vasodilator pathways, the hormone also suppresses the action of vasoconstrictors. Specifically, relaxin treatment markedly attenuates Ang II-induced contraction in the mesenteric artery of diabetic mice (Ng et al., 2017). It is well-established that diabetes leads to an imbalance of vasoactive factors with opposing actions, including NO and Ang II (Tousoulis et al., 2012), favoring vasoconstrictor actions, resulting in impaired vascular homeostasis. Relaxin may have a potential physiological antagonistic effect to the actions of AT-1 or AT-2 angiotensin receptor activation in the mesenteric artery (Marshall et al., 2016, 2017), counteracting diabetes-induced increased in Ang II contraction. It is feasible to speculate that relaxin counter-regulated the AT-1 mediated actions of Ang II by a mechanism which involved increased NO production. Indeed, relaxin treatment has been previously demonstrated to relieve Ang II-induced hypertension by increasing NO availability (Sasser et al., 2011), with possible concomitant reduction of oxidative stress (Sasser et al., 2014).

## Roles of Relaxin in the Treatment of Diabetes-Induced Cardiac Remodeling

Left ventricular hypertrophy and fibrosis are associated with the progression of diabetic cardiomyopathy (Boudina and Abel, 2010). Relaxin treatment for the final 2 weeks of type 1 diabetes results in beneficial cardiovascular effects in diabetic mice, alleviating cardiomyocyte, and pro-hypertrophic gene expression hypertrophy (Ng et al., 2017). Interestingly, the cardioprotective effect of relaxin appears to selectively suppress cardiomyocyte hypertrophy, but not fibrosis, at least in the setting of a relatively short-term 2 weeks of relaxin administration, in direct contrast to the well-documented anti-fibrotic effects of relaxin in other animal models (Samuel et al., 2008, 2011, 2014). Relaxin is thought to inhibit the progression of fibrosis through suppression of TGF-β and downregulation of Smad2 (Samuel et al., 2014) and Smad3 phosphorylation (Sassoli et al., 2013). Given that left ventricular TGF-β gene expression was not elevated in placebo-treated type 1 diabetic mice in the above study (Ng et al., 2017), it is rational to speculate that the ability of relaxin to limit cardiac fibrosis may relate to its distinct action to restore physiological levels of TGF-β (Sassoli et al., 2013). Another possibility is that the anti-fibrotic properties of relaxin may be mediated by its ability to restore physiological levels of the inflammatory cytokine interleukin (IL)-6, as distinct to CTGF/TGF-β pathways, hence resulting in a moderate decrease in diabetes-induced interstitial cardiac collagen content as recently shown (Ng et al., 2017). Such speculation is consistent with that previously shown by Dschietzig et al. (2004), where relaxin suppressed endotoxin-induced increase in IL-6 in THP-1 cells *in vitro* (Dschietzig et al., 2004). Although relaxin fails to reverse diabetes-induced fibrosis in the left ventricle, it elicits potent anti-apoptotic actions in diabetic mouse myocardium (Ng et al., 2017). Consistent with this finding, Moore et al. (2007) also reported that relaxin treatment attenuated hydrogen peroxide-induced apoptosis in neonatal rat cardiomyocytes, an effect attributed to the actions of pro-survival proteins such as Akt and Bcl-2 (Moore et al., 2007). Taken together, relaxin treatment not only shows promising effects in the vasculature, but also in the heart in experimental settings of type 1 diabetes, by alleviating left ventricular hypertrophy and apoptosis.

## Relaxin as an Adjunctive Treatment in Diabetes?

We and others have showed that relaxin has promising therapeutic effects to limit diabetes-induced cardiovascular complications with experimental evidence in *in vitro* and *in vivo* studies, as summarized in **Table 1**. Although the majority of rodent studies have utilized animal models that mimic type 1 diabetes, it remains necessary to determine whether or not relaxin exhibits similar actions in type 2 diabetes, utilizing spontaneously diabetic *db/db* mice or high fat-fed mice. This is important because type 2 diabetes accounts for 85% of patients affected by diabetes. The optimal relaxin treatment period in the context of type 2 diabetes also remains to be determined. In type 1 diabetes which tends to exhibit a juvenile onset, the majority of patients affected are still at a young age at time of diagnosis. Relaxin treatment in humans requires continuous intravenous infusion, hence it is not practical to start relaxin infusion in young children, where prolonged hospitalization might be required. In type 2 diabetes, the proportion of patients affected remain undiagnosed, or are considered pre-diabetic because blood glucose levels are not high enough to be classified as diabetic. Because these patients do not exhibit symptoms of severe diabetes when medical attention would be needed, it would not be feasible to treat with relaxin.

**Table 1:** Cardiovascular effects of relaxin treatment under hyperglycemic conditions.

Duration; dose; route	Vascular effects	References
**6.5 h; 15 μg/h; jugular vein**	Mouse aorta: Increased carbachol-evoked relaxation in lean but not high fat-fed mice.	Bonner et al., 2013
**3 weeks; 1 mg/kg/d; subcutaneous**	Increased carbachol-evoked relaxation in high fat-fed mice.	
**72 h; 10 nM; *in vitro***	Mouse aorta: Prevented high glucose (30 mM)-induced endothelial dysfunction by increasing vasodilator prostacyclin and counteracting superoxide production.	Ng et al., 2016
**2 weeks; 0.5mg/kg/d; subcutaneous**	Mouse aorta: Increased nitric oxide-mediated ACh-evoked relaxation in STZ-induced diabetic mice. Mouse mesenteric artery: Reversed diabetes-induced endothelial dysfunction by increasing nitric oxide-mediated relaxation, normalizing the contribution of vasoconstrictor prostanoids, and reducing vasoconstrictor response to AngII.	Ng et al., 2017
**30 min; 100 ng/mL; *in vitro***	Rat ventricular myocyte: Prevented high glucose (33 mM)-induced apoptosis and endoplasmic reticulum stress by reducing CHOP, cleaved caspase-8, -9, and -12 protein expression.	Zhang et al., 2015
**48 h; 100 ng/mL; *in vitro***	Rat fibroblast: Inhibited high glucose (33 mM)-induced oxidative stress and collagen synthesis by decreasing collagen I and III, α-SMA, P2X7R-mediated NLRP3 inflammasome activation, IL-18, IL-1β, and cleaved caspase-1 expression.	Zhang et al., 2018
**48 h; 0.1 mM; *in vitro***	Rat H9c2 cell line: Reduced high glucose (33 mM)-induced cardiomyocyte hypertrophy, oxidative stress and apoptosis by decreasing ANP, BNP, caspase-3, cytochrome C protein expression, and increasing Notch1, Hes1, and MnSOD expression.	Wei et al., 2018
**72 h; 100 ng/mL; *in vitro***	Rat fibroblast: Reduced high glucose (25 mM)-induced fibroblast proliferation, procollagen I and III, and MMP2 and MMP9 production.	Wang et al., 2009
**2 weeks; 0.5 mg/kg/d; subcutaneous**	Rat left ventricle: Improved diastolic function and decreased myocardial stiffness by reducing α-SMA, TIMP-1 and increasing MMP-13 expression in STZ-induced transgenic mRen-2 rats.	Samuel et al., 2008
**2 weeks; 2 μg/kg/d; subcutaneous**	Rat left ventricle: Improved function by mitigating diabetes-induced apoptosis, fibrosis and inflammasome activation in STZ-induced diabetic rats.	Zhang et al., 2017
**2 weeks; 0.5 mg/kg/d; subcutaneous**	Mouse left ventricle: Suppressed hypertrophy and apoptosis through a reduction in BNP and Bax:Bcl2 expression in STZ-induced diabetic mice.	Ng et al., 2017

*ACh, acetylcholine; AngII, angiotensin II; STZ, streptozotocin; CHOP, C/EBP homologous protein; α-SMA, alpha smooth muscle actin; P2X7R, ionotropic purinergic receptor; NLRP3, nucleotide-binding domain and leucine-rich repeat-containing family, pyrin domain containing 3; IL, interleukin; ANP, atrial natriuretic peptide; BNP, B-type natriuretic peptide; Hes1, hairy and enhancer of split-1; MnSOD, manganese superoxide dismutase; MMP, matrix metalloproteinase; TIMP, tissue inhibitor of metalloproteinase; Bcl2, B-cell lymphoma 2; Bax, Bcl2-associated X protein.*

Although relaxin treatment exhibits fairly profound effects on the vasculature and heart in diabetic mice, its impact on metabolism remains controversial. Relaxin does not alter blood glucose levels, in either healthy rodents (Samuel et al., 2008; Bitto et al., 2013; Bonner et al., 2013; Wong et al., 2013) or in type 1 diabetic models (Samuel et al., 2008; Wong et al., 2013; Ng et al., 2017). However, in type 2 diabetic animal models, relaxin lowers blood glucose levels (Bitto et al., 2013; Bonner et al., 2013). Type 2 diabetes is closely associated with loss of insulin sensitivity and changes in peroxisome proliferator-activated receptor gamma coactivator-1α (PGC-1α) function, particularly at the level of both biogenesis of mitochondria and glucose metabolism (Liang and Ward, 2006). Relaxin modulates PPARγ and PCG-1α signaling pathways, downstream of RXFP1 (Singh et al., 2015). Hence, it is logical to speculate that relaxin may exert metabolic actions in type 2 diabetic mice, modulating their glucose metabolism and/or mitochondria biogenesis. Although there is promising evidence of relaxin blood glucose-lowering actions in mice with type 2 diabetes, it is however important to note that relaxin is ineffective in type 1 diabetes, where insulin administration is required for glycemic control. Hence, relaxin may be an adjunctive agent with glucose-lowering therapy for preventing or treating diabetes. Indeed, relaxin exhibits synergistic effects with the angiotensin converting enzyme inhibitor, enalapril, to abrogate cardiac fibrosis in isoprenaline-induced mouse model (Samuel et al., 2014). Thus, it is possible that relaxin might have similar actions in diabetic mice and could be used in combination with hypoglycemic drugs such as metformin, liraglutide or insulin, to then reduce cardiovascular complications associated with diabetes. However, this hypothesis remains to be tested. It is also yet to be determined whether or not relaxin exhibits adverse effects when used in combination with these conventional anti-diabetic therapies.

## Conclusion

**FIGURE 1:**
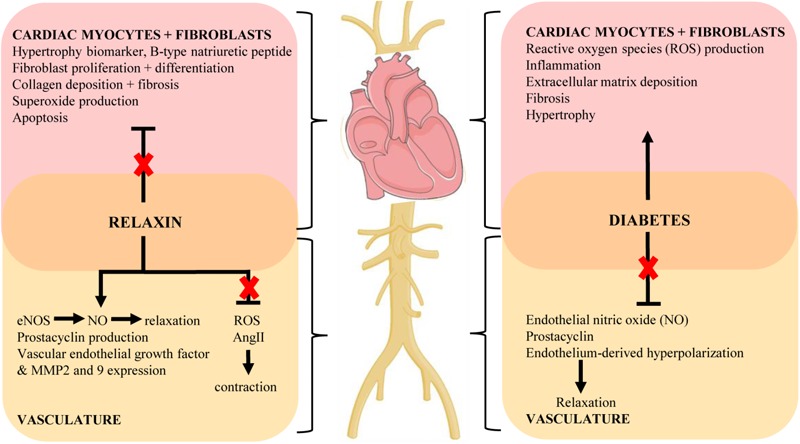
Key mechanisms of the cardiovascular complications of diabetes and proposed protective actions of relaxin to target these pathways (indicated by red cross). Diagram created using artwork provided by Servier Medical Art by Servier, licensed under a Creative Common License.

**Figure 1** shows an overview of the therapeutic potential of relaxin in the pathogenesis of diabetes. Although the conventional glucose-lowering therapies such as metformin and insulin are widely prescribed for the treatment of diabetes, they may not be sufficient to limit the associated cardiovascular complications caused by hyperglycemia. Therefore, we now present the hypothesis that alternative adjunctive agents such as relaxin may offer a promising role in the prevention or treatment of diabetes-related cardiovascular complications, opening up new avenues in addressing the ever-increasing incidence of diabetes and its consequences. Although pre-clinical studies suggest a potential promising role for relaxin to protect against diabetes-associated cardiovascular complications, there remain many unresolved questions relating to the efficacy, dosage and duration of relaxin administration for clinical use in diabetic patients. Clinical trials are needed to specifically determine the translational effects of relaxin in the setting of diabetes, as there are no data available addressing this patient population. As chronic administration of relaxin is not feasible due to its pharmacokinetics and short half-life *in vivo* (Chen et al., 1993a,b), recent development of small molecule relaxin mimetics such as B7-33 (Hossain et al., 2016) and ML290 (Xiao et al., 2013) now warrants such enhanced translation of the vasoprotective effects of relaxin in this setting.

## Author Contributions

HN and RR drafted the manuscript. All authors edited, revised, and approved the final version of this review.

## Conflict of Interest Statement

The authors disclose that this research was partially funded by Novartis Pharma AG, who also provided the serelaxin as a condition of an Australian Research Council Linkage Grant (LP110200543). LP was also a paid consultant for Novartis Pharma AG.
